# Hospital Administration

**Published:** 1894-05-19

**Authors:** W. T. Smedley

**Affiliations:** Honorary Secretary


### The Institutional Workshop.

### HOSPITAL ADMINISTRATION.

### THE BIRMINGHAM HOSPITAL SATURDAY FUND.

By Mr. W. T. Smedley, Honorary Secretary.

Will you allow me to make a few comments on the article on the Birmingham Hospital Saturday Fund which appears in your issue of the 12th inst.? I quite agree with you that the occasion which gives rise to your article was hardly the most suitable one for a discussion on statistics. We had been looking forward to Mr. Burdett's visit to our Hospital Saturday Convalescent Homes, because we recognised his position as the authority on all questions affecting hospitals, and we thought that we should receive the judgment of an expert on what we fondly believed to be the exceptionally good work of the Birmingham movement. We heard with a certain amount of surprise that Mr. Burdett attributed the position of the Birmingham Fund, such as it is, to Mr. Sampson Gamgee, Mr. G. F. Muntz, and Mr. George Dawson. That surprise was intensified when we heard Wolverhampton, West Bromwich, and Coventry held up to us as examples, and when Mr. Burdett exhorted the working men of Birmingham at the forthcoming collection to endeavour to rise to the position which these towns occupied. Dismay followed when we realised what was the effect of bringing an unbiassed, fair, and experienced opinion to bear upon our work. As Mr. Burdett spoke the impression formed upon my mind was that henceforth Birmingham must abstain from comparisons, for in Hospital Saturday matters, either it was lagging so far behind other towns as to make comparisons out of the question, or it was working on lines so distinct from other towns as to make comparisons only misleading.

My connection with the movement has extended over the past twenty-one years, as I took up the work from Mr. Gamgee's hands in 1873, so I may claim to be conversant with its history. Mr. Gamgee was a giant in intellectual attainments, in oratory, and in the power of creating in others a like enthusiasm to that with which he himself was possessed. He carried through the 1873 collection on his broad shoulders. The great success of the inception of the movement was the result of his marvellous power of inspiring others with the enthusiasm he felt. Had he remained its controlling power it would probably to-day have been as far before West Bromwich and Wolverhampton as it now finds itself behind them. When he withdrew from the movement its character was altered, its vitality was gone. The year 1873 was one of exceptional good trade, a fact which greatly assisted the methods of collection which proved so successful, but with declining trade and without Mr. Gamgee's personality the collection sank year after year, until in 1878 £4,705 11s. 3d. had dwindled to £3,134 5s. Then were the original foundations of the movement removed and others substituted. Building operations were commenced on altogether different lines. Mr. Gamgee recognised this and protested. He spoke and wrote against the policy adopted by the movement. On July 27th, 1882, he delivered a public address on "Hospital Saturday: Its Origin and Future; a Glance at Ten Years' Work," in which he strongly criticised the altered conditions under which the collection was conducted. Mr. Sampson Gamgee was undoubtedly the founder of the movement in Birmingham, but whatever success it has attained to-day has not been built on the foundations he laid, but on foundations laid in direct opposition to his advice.

The principle upon which the movement stands to-day is this: (1) That it is the duty of men and women by systematic payments—monthly, fortnightly, or weekly—to make provision by which they can avail themselves of the advantages offered by the hospitals and dispensaries of the city. (2) That it is their duty to contribute towards the cost which the hospitals incur in relieving and healing those who have not the means of providing themselves with passports to the institution whose help they may require—in other words, it is their duty to contribute towards the cost of the free work of the hospitals.

In a manufactory where the weekly system is instituted a committee is formed; the contributions are gathered together. The workpeople become subscribers to the Dispensary, the General Hospital, the Eye Hospital, and any others the tickets for admission to which are likely to be in request. When contributors require admission to the hospitals where registration fees are in force they can obtain the amount from the workshop fund. When Hospital Saturday comes round after purchasing tickets and paying registration fees, there is usually a balance over. This balance is sent as a free gift towards the free work of the hospitals. As a rule it varies between one-third and two-thirds of the total amount subscribed by the workpeople. These surpluses, with the additional amounts collected in manufactories where systematic giving is not in force, amounted in 1893 to £11,328 15s. Such is the Birmingham method—a method which is only applicable to a town where there are ticket hospitals, and one which, as far as I can learn, is carried out nowhere but in Birmingham—a method Mr. Gamgee criticised in the strongest terms, as calculated to take all the virtue out of giving and reduce it to a mere matter of routine.

It must be borne in mind that this weekly payment to the Hospital Saturday collection is not in substitution of the workpeople's sick club in a manufactory. The sick club doctor receives his remuneration from another source. It is to supplement the sick club.

The Birmingham Fund refuses to accept any contribution to which a condition is attached. I have known a cheque for £30 returned to the donors because they stipulated that they should receive tickets for £5 5s. of the amount. "Send us what you like," we said, "keep back five guineas or ten guineas, but what you send must be absolutely free from conditions." The Birmingham Fund is an entity—entirely disassociated from any hospital. It appoints it own officers, manages its own affairs, and the £12,000 which it receives annually is absolutely under the control of the delegates to deal with as they think fit.

In Wolverhampton the Hospital, the Eye Infirmary, and the Hospital for Women fix the same date, and each receives separate collections from the workpeople. There are Hospital Saturday Committees attached to each of these institutions, but their object is to get subscribers to their respective institutions, who shall receive tickets for their subscriptions. The collections, moreover, are managed by the hospital officials. This work corresponds to that portion of the Birmingham Fund the proceeds of which are rigidly excluded from the collection. It is a provident institution—admirable in its way, but as distinct as it is possible to conceive anything in its essential principle from the Birmingham Hospital Saturday collection. In Wolverhampton so much is paid by each manufactory and so many tickets are received in exchange. This is a business transaction creditable to both parties. In Birmingham the money is paid in, and the only return the contributors receive is the consciousness of having discharged an obligation to assist in a noble work. In comparing the two funds I likened the former to copper and the latter to gold. Is my comparison not justified by the facts?

The statement which you attribute to me that the working men contributed £25,000, in addition to the £12,000 Hospital Saturday gift, I certainly never made. You say, "Mr. Smedley further dissented from the statement that £11,000

was the contribution of the working men, and that they received £45,732 worth of benefit for it. He declared indeed, They did not do anything of the sort; they contributed £25,000 to begin with." What I said was, "To begin with, it is £25,000, not £11,000," and I went on to say that in the next place the sum of £45,732 was not expended on Birmingham working men; that the Birmingham hospitals received patients from all parts of the Midland counties; and that the cost of treating these was included in the total of £45,732. I maintain this position still. I certainly never stated that the direct contribution was £25,000, in addition to the free gift of £12,000.

In November, 1892, I published in *Forward*, our Hospital Saturday magazine, an analysis of the sources from which were derived the income of the fifteen medical charities which participate in the collection. The total income was £62,405 7s. 1d.; the expenditure was £55,026 9s. 6d.; whilst the actual expenditure on the relief and maintenance of patients, and administration upon which their awards were made, was £45,971 8s. The income was made up as follows: Musical festival (£5,000), donations sports, theatrical performance (£3,221), legacies (£5,123), sub-lettings, &c.—£14,306 2s. 9d.; dividends, interest, &c., £7,134 7s.; Hospital Sunday, £3,787 13s. 7d.; county subscriptions, £3,573 10s.; total, £28,798 13s. 4d. Nearly the whole of the country subscriptions, it may be said, are given for provident considerations. To the Eye Hospital forty-two Boards of Guardians subscribe £160 per annum; in fact, the country subscriptions of that institution are in excess of the local ones.

This leaves £33,606 13s. 2d. to be accounted for. It is made up thus: Provident subscriptions from workpeoples' societies, £2,001 15s.; provident subscriptions from workpeople, £3,448 7s. 10d.; registration fees, £3,607 17s.; sale of bottles, £116 12s. 4d.—£9,528 9s. Artizans' free gifts: Hospital Saturday, £10,274 16s. 9d.; Sunday-school collection, £258 19s. 1d.; workshop boxes, £64 4s. 6d.—£10,598 0s. 4d., making £20,126 9s. 4d. Subscriptions of firms and private individuals, £13,480 4s. 5d.; total, £33,606 13s. 9d. It will be observed that no credit is taken for the working classes contributing any portion of the Hospital Sunday collection. The Hospital Saturday collection in 1893 was upwards of £1,600 in excess of the amount stated above. The direct subscriptions have increased in a still greater ratio, as have also the registration fees.

The accounts of the medical charities for 1893 have not as yet been issued, but when they are, a complete analysis of them will, I am convinced, show that my statement was correct, and that the workpeople contributed last year £25,000 towards the expenditure of the hospitals.

The discussion which is raised upon the proportion of expenditure which is borne by the working classes opens up a most intricate question, which will probably, at any rate in Birmingham, be brought to the front before long. It is this: What is the position which the hospitals occupy with regard to the public? I remember reading an admirable article in your columns on one aspect of the subject. In it you said, "The hospitals have made the medical profession what it is to-day." It is a pure fiction to suppose that the hospitals exist simply for the benefit of the suffering poor. Every class in the community is personally interested in their efficient maintenance. The well-to-do classes have their own lives and health at stake in the opportunities afforded by them for the practice of medicine and surgery; the working classes here recognise and, I consider, fully discharge their obligations to them. But this is not the time to pursue this subject. If uch a system as you advocate were adopted in Birmingham, that is, if every manufactory was supplied with letters of introduction to the hospitals, which, of course, would give the holder the right to treatment, I believe there would be no difficulty in the working-classes raising from £30,000 to £40,000. But Mr. Burdett commended us for sticking to the free gift system, and urged us to adhere to it whatever we did. To adopt the system which you advocate would surely be to surrender it abjectly.

I am acquainted with the principal Hospital Saturday movements throughout the country. Most of them are in effect provident institutions. Where this is not the case, generally they lack spirit and enthusiasm. Leeds is a notable exception. But I doubt whether anywhere so many men and women take a deep interest in the Hospital Saturday movement as in Birmingham. This interest is growing with an intensity which to those who know its extent and depth is astounding. The scope of the movement is broadening. As a mere organisation it is certainly the most powerful machine which the town has ever seen. We may be wrong, but we do believe that in it and the spirit which has been fostered amongst the workpeople by the belief, it may be a delusion, that their gift is a free one given to help those who are less fortunate than themselves, is to be found one of the most powerful influences for good which exists in our midst.

⁂ We have not space to deal fully with Mr. Smedley's letter this week. He is in error, however, in stating that Hospital Saturday in Wolverhampton is managed by the hospital officials, as it is worked by a Committee on which there are upwards of fifty working men representatives. The registration fees, too, are not working men's contributions nor in any sense provident, but quite the contrary. A registration fee of 1s. paid by an out-patient entails a loss of 2s. 6d. per patient on an average on the hospitals. Birmingham is only like all other large towns in the sense that its hospitals receive patients from outside districts, so no special importance attaches to this fact. The free gifts system, apart from letters of introduction on the Potteries system, must always be delusive and unsatisfactory to all classes.—Ed. *T. H.*

